# Awareness of strengths and weaknesses of cardiovascular magnetic resonance imaging: results from a questionnaire survey

**DOI:** 10.1093/ehjimp/qyae050

**Published:** 2024-05-23

**Authors:** Victoria Zieschang, Rebecca Elisabeth Beyer, Maximilian Leo Müller, Frederike Trautmann, Titus Kühne, Eike Nagel, Andreas Rolf, Andreas Schuster, Grigorios Korosoglou, Henning Steen, Ali Yilmaz, Steffen E Petersen, Bjoern Andrew Remppis, Gisela Thiede, Anna Clara Nolden, Sebastian Kelle

**Affiliations:** Department of Cardiology, Deutsches Herzzentrum der Charité, Angiology and Intensive Care Medicine, Campus Virchow Klinikum, Augustenburger Platz 1, 13353 Berlin, Germany; Charité—Universitätsmedizin Berlin, Corporate member of Freie Universität Berlin and Humboldt-Universität zu Berlin, Charitéplatz 1, 10117 Berlin, Germany; Department of Cardiology, Deutsches Herzzentrum der Charité, Angiology and Intensive Care Medicine, Campus Virchow Klinikum, Augustenburger Platz 1, 13353 Berlin, Germany; Charité—Universitätsmedizin Berlin, Corporate member of Freie Universität Berlin and Humboldt-Universität zu Berlin, Charitéplatz 1, 10117 Berlin, Germany; DZHK (German Centre for Cardiovascular Research), partner site Berlin, Berlin, Germany; Department of Cardiology, Deutsches Herzzentrum der Charité, Angiology and Intensive Care Medicine, Campus Virchow Klinikum, Augustenburger Platz 1, 13353 Berlin, Germany; Charité—Universitätsmedizin Berlin, Corporate member of Freie Universität Berlin and Humboldt-Universität zu Berlin, Charitéplatz 1, 10117 Berlin, Germany; Department of Cardiology, Deutsches Herzzentrum der Charité, Angiology and Intensive Care Medicine, Campus Virchow Klinikum, Augustenburger Platz 1, 13353 Berlin, Germany; Department of Cardiology, Deutsches Herzzentrum der Charité, Angiology and Intensive Care Medicine, Campus Virchow Klinikum, Augustenburger Platz 1, 13353 Berlin, Germany; Institute for Experimental and Translational Cardiovascular Imaging, Goethe University Frankfurt am Main, Frankfurt, Germany; DZHK (German Centre for Cardiovascular Research), partner site Rhine-Main, Germany; Department of Cardiology, Kerckhoff Hospital, University Giessen, Bad Nauheim, Germany; Department of Cardiology and Pneumology, University Medical Center Göttingen, Georg-August University, Göttingen, Germany; Department of Cardiology and Vascular Medicine, GRN Hospital Weinheim, Weinheim, Germany; Weinheim Cardiac Imaging Center, Hector Foundation, Weinheim, Germany; Department of Internal Medicine/Cardiology, Marienkrankenhaus Hamburg, Hamburg, Germany; Department of Cardiology I, Division of Cardiovascular Imaging, University Hospital Münster, Münster, Germany; William Harvey Research Institute, NIHR Barts Biomedical Research Centre, Queen Mary University London, Charterhouse Square, London EC1M 6BQ, UK; Barts Heart Centre, St Bartholomew’s Hospital, Barts Health NHS Trust, West Smithfield, London EC1A 7BE, UK; Department of Cardiology, Heart and Vascular Center Bad Bevensen, Bad Bevensen, Germany; Department of Cardiology, Deutsches Herzzentrum der Charité, Angiology and Intensive Care Medicine, Campus Virchow Klinikum, Augustenburger Platz 1, 13353 Berlin, Germany; Charité—Universitätsmedizin Berlin, Corporate member of Freie Universität Berlin and Humboldt-Universität zu Berlin, Charitéplatz 1, 10117 Berlin, Germany; Department of Cardiology, Deutsches Herzzentrum der Charité, Angiology and Intensive Care Medicine, Campus Virchow Klinikum, Augustenburger Platz 1, 13353 Berlin, Germany; Charité—Universitätsmedizin Berlin, Corporate member of Freie Universität Berlin and Humboldt-Universität zu Berlin, Charitéplatz 1, 10117 Berlin, Germany; Department of Cardiology, Deutsches Herzzentrum der Charité, Angiology and Intensive Care Medicine, Campus Virchow Klinikum, Augustenburger Platz 1, 13353 Berlin, Germany; Charité—Universitätsmedizin Berlin, Corporate member of Freie Universität Berlin and Humboldt-Universität zu Berlin, Charitéplatz 1, 10117 Berlin, Germany; DZHK (German Centre for Cardiovascular Research), partner site Berlin, Berlin, Germany

**Keywords:** cardiac magnetic resonance imaging, CMR, public, awareness, education, survey

## Abstract

**Aims:**

Extensive research has established cardiovascular magnetic resonance (CMR) as a powerful tool for diagnosing and monitoring various cardiovascular diseases (CVDs). However, CMR has yet to reach its full potential in routine clinical care, which is mainly due to reimbursement issues. Among other factors, overcoming this gap requires adequate awareness among healthcare professionals and potential patients, the extent of which is currently unknown. Therefore, we conducted a survey to assess awareness and identify knowledge gaps regarding the clinical role and socio-economic factors associated with CMR.

**Methods and results:**

One hundred forty-four subjects not involved in direct patient care were enrolled at a German health conference and completed a 24-item survey, including procedural, clinical, and socio-economic questions about CMR. Respondents were well aware of the socio-economic impact of CVD. Common CMR indications were correctly identified by most participants, but only 22.9% knew the full spectrum. Participants underestimated the modality’s benefits, such as absence of ionizing radiation and rare allergic reactions to contrast agents (only 70.9% and 37.6% correct answers, respectively). Respondents estimated the therapeutic guidance of CMR to be high (50.7% voted impact > 50%) and the annual demand to be increasing (89.9%). Attitudes towards CMR were generally positive, with 77.1% of participants willing to travel >25 km and 60.4% willing to pay >125 Euros to have a CMR examination.

**Conclusion:**

Despite great interest in CMR, significant knowledge gaps hinder its optimal use in clinical practice. The development and implementation of awareness and education strategies are needed to realize the full clinical potential of CMR.

## Lay Summary

Cardiovascular magnetic resonance (CMR) imaging is a highly accurate method for diagnosing and monitoring various heart diseases. However, it is still underused in routine clinical practice. To investigate whether this is due to a lack of awareness, we conducted a survey of both healthcare professionals and potential patients. Our survey showed that, while most respondents were aware of the significant impact of heart disease on society, only a small proportion were fully aware of the indications for and benefits of CMR. Despite these knowledge gaps, we found a generally positive attitude towards CMR, with most respondents being aware of the increasing demand for CMR and recognizing its important role in guiding treatment decisions. In addition, the majority of respondents was willing to travel and make a financial contribution to obtain a CMR scan. Taken together, our findings indicate a strong interest in CMR, but also point to the need for better education and awareness of both healthcare professionals and the public. Addressing the knowledge gaps identified may help to integrate CMR into health care more effectively.

## Graphical Abstract

**Graphical Abstract:**
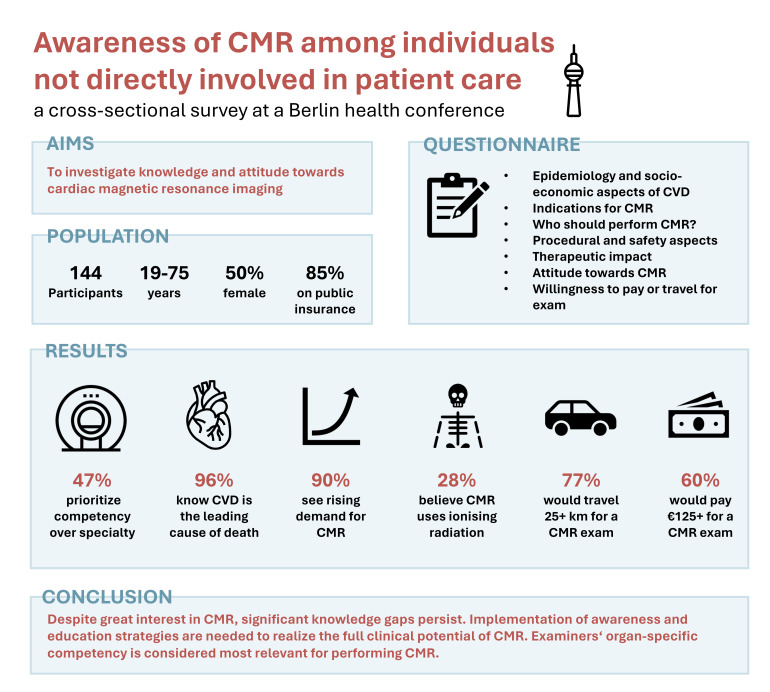

## Introduction

Cardiovascular diseases (CVDs) are the leading cause of mortality worldwide, including in Germany, where it accounted for 33.6% (*n* = 358 219) of all deaths in 2022.^1,2^ Furthermore, CVD is the largest contributor to healthcare expenditure in Germany, accounting for 13.7% (€46.4 billion) of total costs in 2015.^3^

Irrespective of the underlying aetiology, all CVDs can lead to heart failure (HF).^4^ Over the past decades, cardiovascular magnetic resonance (CMR) imaging has emerged as a powerful tool for the diagnosis, assessment, and monitoring of HF, both in research and in clinical care.^5^ In fact, assessment by CMR is recommended by the current HF guidelines of the European Society of Cardiology and the American Heart Association, especially for the evaluation of specific infiltrative and inflammatory cardiomyopathies.^6–8^

Accordingly, the demand for CMR has increased steadily in recent years.^9^ Nevertheless, CMR has yet to reach its full potential in clinical care. Among other reasons, this may be due to a lack of awareness and understanding of the clinical role of CMR and associated socio-economic factors among healthcare professionals, associated industry, and potential patients. In Germany, like in most other European countries, reimbursement issues have yet to be resolved. Raising awareness for the clinical and prognostic impact may be the most effective step to overcome this issue, as there is also a huge body of evidence pertaining to the socio-economic potential of the method.

This study aims to report on the awareness of and identify knowledge gaps regarding the clinical role and socio-economic factors associated with CMR among healthcare staff and allied professionals. The results of this study are expected to provide valuable insights for healthcare providers, help identify ongoing gaps in patient education, and inform future strategies to make CMR more accessible to eligible patients.

## Methods

A cross-sectional survey was conducted at the ‘Hauptstadtkongress 2019—Medizin und Gesundheit’ (HSK) in Berlin between 21 and 23 May 2019.

The study included a random sample of visitors and exhibitors at the HSK. All participants provided written informed consent to participate in this study. Participants received a paper-based, self-administered 24-item CMR-focused questionnaire, which was developed by members of the MRI Core Lab Berlin and edited by cardiology and CMR experts. In addition to socio-demographic questions (e.g. gender, age, occupation, and insurance status) and general questions about the role of CVD in the German healthcare system, the questionnaire covered procedural, medical, and financial aspects of CMR (e.g. patient eligibility and indications, requirements for CMR procedure and duration of examination, comparison with other imaging modalities, and impact on treatment). Additionally, the survey included three questions about the participants’ attitudes towards CMR. The questionnaire was administered in German by a team of medical students and physicians. Participation in the survey was voluntary, confidential, and anonymous, and no incentives were offered for responding. A translated version of the full questionnaire can be found in the Appendix.

For analysis, participants were divided into two subgroups according to their occupation (i.e. participants involved in direct patient care vs. participants not involved in direct patient care). In the following data of the subgroup that was not directly involved in patient care will be presented. Data on the subgroup of participants directly involved in patient care can be found in the Supplementary data. Categorical data are presented as numbers and percentages. Continuous data are presented as mean with standard deviation (SD). Individual questions were excluded from the analyses if answers were missing or if multiple answers were selected where only one was required. Statistics were performed with R version 4.3.0 (The R Foundation for Statistical Computing, Vienna, Austria). Statistical significance was assumed at a two-sided *P*-value < 0.05.

## Results

A total of 190 attendees participated in the survey. Based on their self-reported occupation, 39 respondents (20.5%) were assigned to the ‘participants involved in direct patient care’ subgroup and 144 participants (75.7%) were assigned to the ‘participants not involved in direct patient care’ subgroup. The remaining seven participants (3.8%) did not report their occupation and were therefore not assigned to any of the subgroups. The demographic characteristics of the participants not directly involved in patient care are summarized in *Table 1*. Unless otherwise stated, the terms ‘participants’ or ‘respondents’ in the following sections refer to individuals not directly involved in patient care. Overall, 50% of participants were female with a mean age of 39 ± 14 years. A total of 15% of participants were privately insured.

**Table 1:** Demographic characteristics of the surveyed participants who were all not directly involved in patient care

	Participants not involved in direct patient care (*n* = 144)
Age (years)	39 (±14)
Sex	
Male	69 (50%)
Female	68 (50%)
Health insurance	
Statutory health insurance	109 (85%)
Private health insurance	19 (15%)
Profession	
Administration	31 (22%)
Industry	27 (19%)
IT	12 (8.3%)
Health insurance company	8 (5.6%)
Politics	7 (4.9%)
Health association	7 (4.9%)
Other professions	52 (36%)
Not indicated	—

Continuous data are presented as mean with standard deviation (SD). Categorical data are presented as counts with corresponding percentages. *P*-values are derived from comparisons between subgroups.

Summarized responses to selected questions from the survey are presented in *Table 2*.

**Table 2:** Overview of answers to selected questions for participants not involved in direct patient care

Question	Participants not involved in direct patient care (*n* = 144)
Which of the following is the leading cause of death in Germany?	
Breast cancer	1 (0.7%)
Respiratory diseases	1 (0.7%)
*Cardiovascular diseases*	136 (95.8%)
Dementia	0 (0%)
Intestinal cancer	3 (2.1%)
Which of the following causes the highest annual healthcare expenditure in Germany?	
Cancer	48 (33.8%)
*Cardiovascular diseases*	89 (62.7%)
Respiratory diseases	5 (3.5%)
Which of the following examinations exposes patients to radiation? (Multiple answers allowed)	
*Cardiac CT*	96 (68.1%)
Cardiac MRI	39 (27.7%)
Cardiac ultrasound/echocardiography	29 (20.6%)
*Cardiac scintigraphy*	57 (40.4%)
*Cardiac catheterization*	38 (27.0%)
What is the maximum body weight up to which a cardiac MRI exam can technically be carried out using modern devices?	
80 kg	2 (1.4%)
100 kg	11 (7.7%)
125 kg	33 (23.1%)
150 kg	53 (37.1%)
*>180* *kg*	44 (30.8%)
How long does a cardiac MRI exam usually take?	
10–20 min	66 (47.8%)
30 min	30 (21.7%)
60 min	7 (5.1%)
*Variable (15–60 min)*	35 (25.4%)
Which medication is mandatory during a cardiac MRI exam? (Multiple answers allowed)	
*None*	87 (62.1%)
Contrast agent	57 (40.7%)
Pharmaceutical stress agent	11 (7.9%)
Pain killers	3 (2.1%)
Tranquilizers	20 (14.3%)
Who should carry out and evaluate a cardiac MRI exam?	
Only radiologists	30 (21.7%)
Only cardiologists	46 (33.3%)
*Not important if examiner is board certified*	65 (47.1%)
Which of the following is a/are valid indication(s) for a cardiac MRI examination? (Multiple answers allowed)	
*Suspected perfusion deficit of the heart muscle*	103 (73.0%)
*Unclear thickening of the heart muscle*	120 (85.1%)
*Impaired pumping function/weakness of the heart*	87 (61.7%)
*Infections/inflammation of the heart muscle*	81 (57.4%)
*Evaluation before surgery/evaluation of surgical risk*	82 (58.2%)
*Inherited diseases of the heart muscle (‘Cardiomyopathies’)*	88 (62.4%)
*Congenital heart diseases*	103 (73.0%)
Which of the following factors would you consider as advantages of cardiac MRI? (Multiple answers allowed)	
*No radiation exposure*	100 (70.9%)
*Cost-effectiveness*	13 (9.2%)
Easily available	33 (23.4%)
*Rare allergic reactions to contrast agents*	53 (37.6%)
*Good image quality/informative value, independent of the patient’s constitution*	91 (64.5%)
*High temporal and spatial resolution*	53 (37.6%)
*Reproducible, regardless of the examiner’s experience*	49 (34.8%)
How would you rate the predictive power of a suspicious stress perfusion cardiac MRI for a future heart attack in comparison to a suspicious stress echocardiogram?	
*Cardiac MRI is better*	91 (64.5%)
Same	19 (13.5%)
Cardiac MRI is worse	3 (2.1%)
I do not know	28 (19.9%)
How would you rate the diagnostic accuracy of cardiac MRI for coronary artery disease compared with a myocardial perfusion scintigraphy?	
*Cardiac MRI is better*	53 (38.1%)
Same	31 (22.3%)
Cardiac MRI is worse	4 (2.9%)
I do not know	51 (36.7%)
In how many cases do the results from a cardiac MRI exam have an influence on the therapy of the patient?	
Never	9 (6.3%)
In 20% of the cases (every fifth examination)	31 (21.8%)
In 30% of the cases	30 (21.1%)
*In 50% of the cases*	43 (30.3%)
In 70% of the cases	29 (20.4%)
Which health insurance currently reimburses the costs for a cardiac MRI exam in Germany?	
*Only private health insurance companies*	27 (19.6%)
Only statutory health insurance companies	9 (6.5%)
Both	102 (73.9%)
How would you assess the development of current demand for cardiac MRI?	
Decreasing	0 (0%)
Constant	14 (10.1%)
*Annual increase around 10%*	119 (85.6%)
Annual increase around 50%	6 (4.3%)
How far (from your hometown) would you be willing to travel to obtain a cardiac MRI exam? (Multiple answers allowed)	
Only if it is available in my hometown	18 (12.5%)
Up to 10 km	6 (4.2%)
Up to 25–50 km	61 (42.4%)
Up to 100 km	50 (34.7%)
I would prefer a mobile cardiac MRI exam (exam in my hometown and evaluation by experts via telemedicine)	24 (16.7%)
How far (from your hometown) would you be willing to travel to obtain a cardiac MRI exam? (Multiple answers allowed)	
75 Euro	50 (39.7%)
125 Euro	39 (31.0%)
250 Euro	28 (22.2%)
500 Euro	7 (5.6%)
1000 Euro	2 (1.6%)

The frequency of given answers is provided as count with corresponding percentage of evaluable replies within the respective question. Correct answers (if applicable) are written in italics.

### Socio-economic importance of CVD

Regarding the medical and socio-economic impact of CVD in Germany, 95.8% of respondents correctly identified CVD as the leading cause of death and 62.7% knew that CVDs account for higher annual healthcare costs than cancer or respiratory diseases.

### General knowledge about CMR

**Figure 1:**
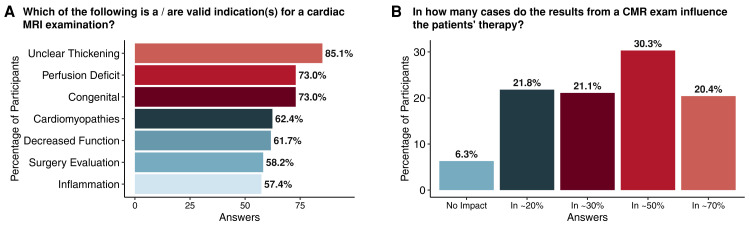
Knowledge and awareness of indications (*A*) and therapeutic impact (*B*) of cardiac MRI among the participants who are not involved in direct patient care.

Participants were then asked which clinical scenarios they would consider to be valid indications for a CMR scan. The responses are summarized in *Figure 1A*. ‘Unclear thickening of the heart muscle’ was the most common response (85.1%), followed by ‘suspected perfusion deficit of the heart muscle’ (73%), and ‘congenital heart disease’ (73%). Slightly less than a quarter (22.9%) of all respondents were able to correctly identify all possible CMR indications.

Regarding technical aspects, the majority of respondents (62.1%) knew that no specific medication is required to perform a CMR scan. Only 30.8% of respondents correctly answered that with modern equipment, CMR exam can be performed even in patients with a body weight > 180 kg. Most respondents (37.1%) thought that 150 kg was the maximum body weight up to which a CMR scan was technically possible.

Regarding inherent risks, 27.7% incorrectly believed that CMR scans are associated with radiation exposure. The same was true for echocardiography (20.6%). In contrast, 68.1%, 40.4%, and 27% of respondents were aware of ionizing radiation exposure during cardiac CT, cardiac scintigraphy, and cardiac catheterization, respectively.

From a list of advantages of CMR, ‘no radiation exposure’ (70.9%) and ‘good image quality/informative value independent of the patient’s constitution’ (64.5%) were the answers with the highest number of votes, followed by ‘high temporal and spatial resolution’ and ‘rare allergic reactions to contrast agents’ with 37.6% each. The lowest number of votes felt that advantages of CMR are to be ‘easily available’ (23.4%) or ‘cost-effective’ (9.2%), respectively.

When asked to rate the predictive power of a suspicious stress test, 64.5% of participants correctly rated CMR as superior to echocardiography, while only 2.1% of respondents thought that echocardiography was superior. Similarly, the most common response (38.1%) was that CMR has a higher diagnostic accuracy for coronary artery disease than myocardial perfusion scintigraphy. However, in another question (*Figure 1B*), 49.2% of respondents underestimated the clinical impact of a CMR scan on the subsequent choice of therapy. Specifically, only 30.3% knew that the results of a CMR scan influence the choice of therapy in ∼50% of cases, and 6.3% even believed that they have no influence at all. In contrast, 20.4% overestimated the impact of CMR on clinical decision-making and chose the answer ‘in ≈70% of cases’.

### Cost, demand, and availability of CMR

**Figure 2:**
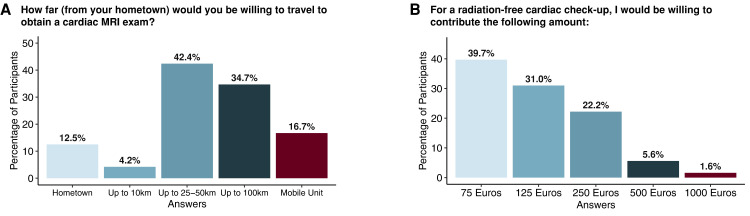
Participants’ (not involved in direct patient care) willingness to travel (*A*) and financially contribute (*B*) to obtain a cardiac MRI examination.

Regarding the current demand for CMR examinations, 85.6% of respondents correctly answered that it is increasing at an annual rate of ∼10%, and 4.3% of respondents even considered an annual increase of ∼50% to be most likely. The majority of participants (73.9%) incorrectly believed that the cost of a CMR scan is currently reimbursed regardless of the type of health insurance, whereas only 19.6% knew that reimbursement is limited to private health insurance companies in Germany. Notably, more than half of the respondents (60.4%) stated that they would be willing to pay 125 Euros or more for a radiation-free cardiac check-up, and more than a quarter (29.4%) would pay 250 Euros or more (*Figure 2B*). Moreover, 42.4% of participants would be willing to travel 25–50 km for a CMR scan, and 34.7% would even be willing to travel up to 100 km. A total of 16.7% of respondents specifically favoured a mobile CMR examination near their hometown with telemedical supervision by experts (*Figure 2A*).

## Discussion

To our knowledge, this study is the first to assess the awareness of CMR in Germany. Our results reveal a significant gap in the general knowledge and perception of CMR among participants who are not involved in direct patient care, despite its growing importance in cardiovascular medicine.^6–8,10^ While most study participants correctly identified CVD as the leading cause of death (95.8% correct answers) and the main contributor to healthcare expenditure in Germany (62.7% correct answers), our data reveal a surprising discrepancy in their understanding of the related diagnostic imaging modalities such as CMR.^1–3^

### General knowledge of CMR

Our survey discloses a heterogeneous awareness of the role of CMR in the diagnostic work-up of various CVDs, although the list of indications for which CMR can be used is constantly expanding.^6–8,10^ While some indications, such as ‘unclear thickening of the heart muscle’ (85.1%), ‘suspected perfusion deficit’ (73%), and ‘congenital heart disease’ (73%), were familiar to most respondents in our survey, only less than a quarter of our respondents (22.9%) were able to identify all possible indications that might be of interest to them or their patients. Notably, we did not observe a significant difference in the awareness of possible indications for CMR between participants involved in direct patient care (data provided in Supplementary data online, *Table S2*) and participants not involved in direct patient care.

In terms of procedural risks, 27.7% of the respondents incorrectly identified CMR as being associated with ionizing radiation, which is consistent with previous research.^11,12^ For example, Ria *et al*.^11^ previously found that 37.5% of their patients believed that MRI scans involved exposure to ionizing radiation. The lower proportion in our survey is likely to be influenced by selection bias, as our participants were recruited at a health conference, suggesting an interest in healthcare-associated topics. However, it should be noted that even among the respondents who identified themselves as participants involved in direct patient care in our study (see Supplementary data), 18.4% believed that CMR exposed their patients to radiation and only 79.5% correctly identified ‘no radiation exposure’ as an advantage of CMR. Considering that another 20.6% of participants not directly involved in patient care responded that echocardiography is associated with ionizing radiation, further education is urgently needed. Apart from the quality of the test, the risks associated with the procedure constitute a criterion that strongly influences the choice of diagnostic and therapeutic procedures made by providers and patients. It has been shown that healthcare providers are more likely to opt for MRI rather than CT when informed about the associated radiation exposure.^13^ Therefore, education about CMR as a radiation-free and low-risk modality is crucial to establish it as an equivalent alternative to echo and to realize its full diagnostic potential.

We suspect that most respondents not directly involved in patient care were unaware of the differences between Gadolinium-based contrast agents (GBCAs) for MRI and iodine-based contrast agents for computed tomography (CT), and therefore did not know that one of the advantages of CMR is that second generation GBCAs, in particular, have a very low incidence of adverse reactions (0.4/1000 doses vs. 1.5/1000 doses for iodine-based contrast agents for CT).^14^ A fact that was much more commonly known among participants directly involved in patient care (56.4%, *P* = 0.035, see Supplementary data online, *Table S2*).

Study participants believed that severe obesity might be a factor making CMR examinations technically infeasible. However, using a large US registry (*n* = 2345), Ge *et al*.^15^ demonstrated that the diagnostic quality and predictive power of stress CMR are maintained regardless of body mass index (BMI). Previously published data from our own centre (*n* = 2585) even suggested that the prognostic value of non-suspicious dobutamine stress CMR is higher in obese (BMI ≥ 30) than in overweight or normal weight patients.^16^ In line with these findings, it has been claimed that CMR is probably the best choice of non-invasive imaging modality for detecting coronary artery disease in obese patients and several centres located in the USA claim that, using modern scanners with a bore diameter of 70 cm can scan patients with a body weight of >200 kg.^17,18^ Thus, educating medical staff and patients that CMR is technically feasible and has sustained diagnostic value even in severely obese individuals is particularly important to improve the uptake of CMR in routine clinical care, as obesity is already a pressing and yet growing public health problem.

In general, participants underestimated the length of a CMR scan. Although improved techniques such as three-dimensional ultrafast scanning (median door-to-door time of 11 min and 52 s) or rapid assessment of suspected coronary artery disease (<20 min) have been shown to be feasible while maintaining good image quality, they are not standard practice in most clinics and do not reflect clinical reality.^19^

More importantly, however, respondents also underestimated the impact of CMR results on clinical decision-making, despite extensive evidence of its substantial impact.^20–22^ Specifically, using data from the European CMR Registry (*n* = 27 781), Bruder *et al*.^22^ demonstrated that CMR had a therapeutic impact in 53.4% of patients regardless of indication, and in as many as 66% of patients undergoing CMR examination for the assessment of myocardial viability. Additionally, Abbasi *et al*.^21^ showed that CMR led to a change in clinical management in 52% of patients with HF and even led to a completely new diagnosis in 30% of cases. In light of these data demonstrating the high diagnostic accuracy of CMR and the clinical reliability of the CMR-guided diagnoses, the identified lack of awareness for the clinical impact of a CMR exam is another important knowledge gap that needs to be filled, especially among healthcare professionals referring patients for a diagnostic work-up of suspected CVD, in order to realize the full potential of CMR.

### Perceptions and expectations towards CMR

Although there appear to be significant gaps in knowledge about CMR, the present data highlight that participants not involved in direct patient care generally have a positive perception of CMR and high expectations of it. This is reflected by the fact that 89.9% of respondents described the current demand for CMR testing as increasing, with 85.6% correctly estimating the annual increase in demand to be around 10%, as has been shown by Asher *et al*.^23^ Interestingly, 23.1% participants directly involved patients care (see Supplementary data) estimated annual demand to rising by a number as high as 50% per year, potentially reflecting the increasingly important role that CMR plays in first-line work-ups and routine clinical decision-making. Our data can also be interpreted to mean that this demand is not currently being met, with only 23.4% of respondents believing that CMR is readily available. However, participants were surprisingly willing to travel to overcome this shortage. Specifically, 77.1% responded that they would travel more than 25 km, with 34.7% indicating that they would even be willing to travel up to 100 km to obtain a CMR exam. Taken together, these findings suggest that a lack of availability is another factor that should be addressed to maximize the clinical utility of CMR. However, it does not appear necessary for the equipment and trained staff to be available in every medical facility. Instead, an even distribution across cities and larger district towns may be sufficient to be able to offer CMR to most patients. More modern and unconventional approaches, such as a mobile CMR unit with telemedical supervision by experts from established CMR centres, may be an alternative, as evidenced by the fact that 16.7% of respondents in our survey expressed a preference for this option. The concept of a mobile CMR unit has previously been shown to work, even when performing adenosine stress tests, but further research is needed to assess the technical feasibility, safety, clinical indications and associated costs on a larger scale.^24^

Regarding cost, only 9.2% of respondents in our survey perceived CMR examinations to be cost-effective, although the latter has already been demonstrated in several studies in patients with (suspected) coronary artery disease.^25–27^ A recent paper, Pandya and colleagues were able to review the literature on the cost-effectiveness of CMR in coronary artery disease (CAD) and create a globally applicable cost-effectiveness calculator to estimate the cost per quality-adjusted life year of CMR and relevant comparators with context-specific patient- and system-level inputs based on current global evidence in the literature. They demonstrated that CMR using different scenarios is generally a cost-effective option compared with relevant comparators for detecting significant CAD.^28^

**Figure 3:**
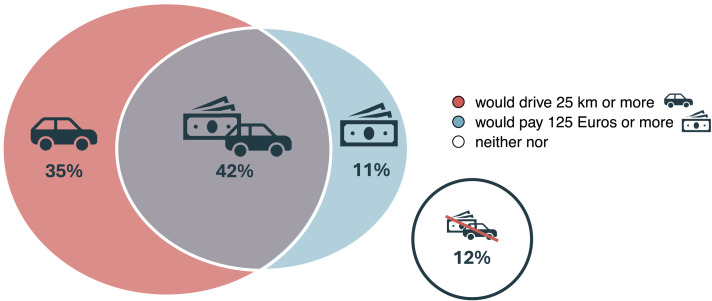
Venn diagram illustrating the overlap of participants’ (not involved in direct patient care) responses regarding their willingness to travel and financially contribute to obtain a CMR exam.

Interestingly, the majority of respondents in our survey (60.4%) indicated that they would be willing to provide a financial contribution of more than 125 Euros for a CMR examination, highlighting the perceived benefit of CMR. In total, 43% of respondents indicated that they would be prepared to travel more than 25 km for a radiation-free scan and also be prepared to make a co-payment of 125 Euros or more (*Figure 3*). Such financial contributions could help to make CMR available to patients with statutory health insurance (who make up 85% of our study sample), as currently CMR scans are only reimbursed by private health insurance companies in Germany, a fact that only 19.6% of respondents to our survey were aware of.

**Figure 4:**
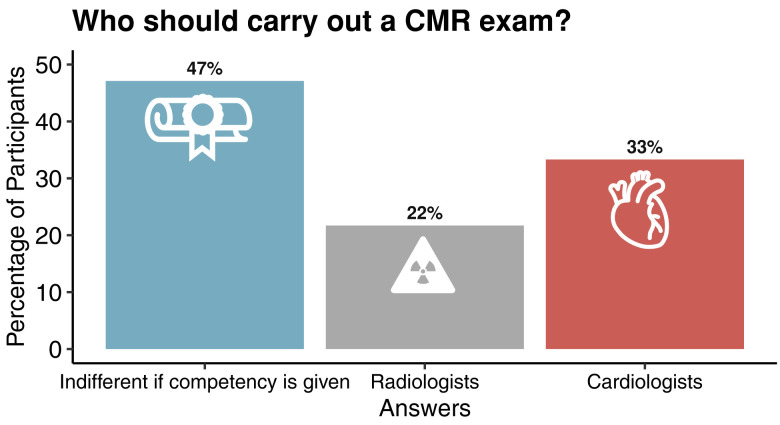
Perception of who should be carrying out their CMR exam and interpretation among survey participants’ who are not involved in direct patient care.

With an ever-increasing demand and recent incorporation of CMR as first-line imaging modality in the ESC guidelines,^29^ there is great need for more trained professionals carrying out CMR exams. However, varying guidelines and curricula across countries and societies, as well as the possibility to perform and report a CMR scan by either radiologists or cardiologists, still represent a limitation to CMR quality and diffusion across many European countries.^30,31^ Recent statements by the SCMR and the joint ESC taskforce advocated for establishing competency-based, standardized guidelines and certification processes for uniform global application and training regardless of the examiner’s speciality.^30,31^ This is supported by our findings (*Table 3*, *Figure 4*), which show that in the eye of participants who are not involved in direct patient care—who themselves may become patients—high-quality training and examiner’s experience in the relevant clinical field are perceived as the most essential factor for safe and insightful CMR.

**Table 3:** Overview of answers to the question regarding preferred examiner for the entire cohort, as well as subgroups containing participants involved in direct patient care and participants not involved in direct patient care

	Who should carry out and evaluate a cardiac MRI exam?			
	Entire cohort	Participants involved in direct patient care	Participants not involved in direct patient care	*P*-value
Only radiologists	41 (22.7%)	9 (24.3%)	30 (21.7%)	0.7
Only cardiologists	61 (33.7%)	11 (29.7%)	46 (33.3%)	0.7
*Not important if examiner is board certified*	82 (45.3%)	17 (45.9%)	65 (47.1%)	0.9

Participants not reporting an occupation (n = 7) were included in the entire cohort but not in any of the subgroups. The frequency of given answers is provided as count with corresponding percentage of evaluable replies within the respective (sub)group. Correct answers (if applicable) are written in italics. P-values are derived from comparisons of the two subgroups and indicate statistical significance if <0.05.

### Limitations

Our study sample was derived from participants at a health and medical event in Berlin (HSK), which may introduce a selection bias and limit the generalizability of our findings to the general population. Another important limitation of our survey is that it does not allow for a fully precise description of the participants’ profession and previous experience (either in the form of specialized training or as a patient) with CMR. Specifically, when asked for their profession, a significant proportion of respondents indicated ‘others’ but did not provide any further specification, introducing some heterogeneity into the ‘participants not involved in direct patient care’ group. A question concerning previous experience with CMR as a patient, which may have provided insights into how this affects awareness for the modality’s benefits, was missing. Furthermore, our questionnaire did not differentiate between doctors, nurses, and medical technicians within the ‘participants involved in direct patient care’ subgroup (results reported in Supplementary data online, *Tables S1* and *S2*). As a result, the awareness of CMR among referring physicians may have been underestimated.

## Conclusion

The results of our survey highlight a paradox in the public perception of CMR in Germany: although there is a high level of interest and diagnostic value placed on the modality, significant knowledge gaps remain. This disparity calls for strategic education and awareness initiatives across multiple channels to improve the understanding of CMR among participants involved in direct patient care and participants not involved in direct patient care as well as potential patients. Improved awareness could lead to better funding, reimbursement policies, and broader integration of CMR into diagnostic algorithms, ultimately benefiting patients in routine clinical care.

## Supplementary data

Supplementary data are available at *European Heart Journal – Imaging Methods and Practice* online.

## Supplementary Material

qyae050_Supplementary_Data

## Data Availability

The data underlying this article will be shared on reasonable request to the corresponding author.

## Lead author biography

Victoria Zieschang (MD), who completed her medical studies at the Charité University in Berlin in 2023, is currently working as a radiology resident at the Charité Hospital in Berlin. Her academic and professional career has started with focused research in the area of cardiac MRI analysis, carried out at the German Heart Center Berlin, Germany, where she was part of MRI-Core-Lab/working group on CMR at the Department of Internal Medicine/Cardiology. In addition, Victoria Zieschang has used her expertise to investigate the use of cardiac MRI in congenital heart disease in children.

## References

[1] (WHO) WHO. Cardiovascular diseases (CVD): World Health Organization (WHO). https://www.who.int/news-room/fact-sheets/detail/cardiovascular-diseases-(cvds) (2 May 2024, date last accessed).

[2] (DESTATIS) DSB. Todesursachen in Deutschland: Deutsches Statistisches Bundesamt (DESTATIS). 2024. https://www.destatis.de/DE/Themen/Gesellschaft-Umwelt/Gesundheit/Todesursachen/_inhalt.html; jsessionid=E1903F30B462F31C4230322D4B203A48.internet8731#235880 (2 May 2024, date last accessed).

[3] (DESTATIS) DSB. Herz-Kreislauf-Erkrankungen verursachen die höchsten Kosten: Deutsches Statistisches Bundesamt (DESTATIS). 2017. https://www.destatis.de/DE/Presse/Pressemitteilungen/2017/09/PD17_347_236.html (2 May 2024, date last accessed).

[4] Ziaeian B, Fonarow GC. Epidemiology and aetiology of heart failure. Nat Rev Cardiol 2016;13:368–78. 26935038 10.1038/nrcardio.2016.25 PMC4868779

[5] Petersen S, Ludman A, Davies L. CMR in Heart Failure: Current and Emerging Clinical Applications. Latest Advances in Clinical and Pre-Clinical Cardiovascular MRI. United Arab Emirates: Bentham Science Publishers; 2015.

[6] McDonagh TA, Metra M, Adamo M, Gardner RS, Baumbach A, Bohm M et al 2021 ESC guidelines for the diagnosis and treatment of acute and chronic heart failure. Eur Heart J 2021;42:3599–726. 34447992 10.1093/eurheartj/ehab368

[7] Heidenreich PA, Bozkurt B, Aguilar D, Allen LA, Byun JJ, Colvin MM et al 2022 AHA/ACC/HFSA guideline for the management of heart failure: a report of the American College of Cardiology/American Heart Association Joint Committee on Clinical Practice Guidelines. Circulation 2022;145:e895–1032. 35363499 10.1161/CIR.0000000000001063

[8] Arbelo E, Protonotarios A, Gimeno JR, Arbustini E, Barriales-Villa R, Basso C et al 2023 ESC guidelines for the management of cardiomyopathies. Eur Heart J 2023;44:3503–626. 37622657 10.1093/eurheartj/ehad194

[9] Ripley DP, Musa TA, Dobson LE, Plein S, Greenwood JP. Cardiovascular magnetic resonance imaging: what the general cardiologist should know. Heart 2016;102:1589–603. 27559093 10.1136/heartjnl-2015-307896

[10] von Knobelsdorff-Brenkenhoff F, Schulz-Menger J. Cardiovascular magnetic resonance in the guidelines of the European Society of Cardiology: a comprehensive summary and update. J Cardiovasc Magn Reson 2023;25:42. 37482604 10.1186/s12968-023-00950-z PMC10364363

[11] Ria F, Bergantin A, Vai A, Bonfanti P, Martinotti AS, Redaelli I et al Awareness of medical radiation exposure among patients: a patient survey as a first step for effective communication of ionizing radiation risks. Phys Med 2017;43:57–62. 29195563 10.1016/j.ejmp.2017.10.014

[12] Ribeiro A, Husson O, Drey N, Murray I, May K, Thurston J et al Ionising radiation exposure from medical imaging—a review of patient’s (un) awareness. Radiography (Lond) 2020;26:e25–30. 32052780 10.1016/j.radi.2019.10.002

[13] Gimbel RW, Fontelo P, Stephens MB, Olsen CH, Bunt C, Ledford CJ et al Radiation exposure and cost influence physician medical image decision making: a randomized controlled trial. Med Care 2013;51:628–32. 23604013 10.1097/MLR.0b013e3182928fd5

[14] Morzycki A, Bhatia A, Murphy KJ. Adverse reactions to contrast material: a Canadian update. Can Assoc Radiol J 2017;68:187–93. 27745988 10.1016/j.carj.2016.05.006

[15] Ge Y, Steel K, Antiochos P, Bingham S, Abdullah S, Mikolich JR et al Stress CMR in patients with obesity: insights from the Stress CMR Perfusion Imaging in the United States (SPINS) registry. Eur Heart J Cardiovasc Imaging 2021;22:518–27. 33166994 10.1093/ehjci/jeaa281 PMC8599869

[16] Kelle S, Giusca S, Buss SJ, Fleck E, Katus HA, Korosoglou G. BMI does not influence the prediction of cardiac events using stress CMR. Int J Cardiol 2015;179:31–3. 25464402 10.1016/j.ijcard.2014.10.064

[17] Bigvava T, Zamani SM, Pieske-Kraigher E, Gebker R, Pieske B, Kelle S. Prognostic value of non-invasive stress testing for coronary artery disease in obese patients. Expert Rev Cardiovasc Ther 2015;13:1325–32. 26536394 10.1586/14779072.2015.1102054

[18] University of California SF. Size and Weight Considerations for Imaging and Image-Guided Interventions: University of California, San Francisco. https://radiology.ucsf.edu/patient-care/patient-safety/size-weight#accordion-mri-scanners (2 May 2024, date last accessed).

[19] Gomez-Talavera S, Fernandez-Jimenez R, Fuster V, Nothnagel ND, Kouwenhoven M, Clemence M et al Clinical validation of a 3-dimensional ultrafast cardiac magnetic resonance protocol including single breath-hold 3-dimensional sequences. JACC Cardiovasc Imaging 2021;14:1742–54. 33865783 10.1016/j.jcmg.2021.02.031 PMC8421247

[20] Rajwani A, Stewart MJ, Richardson JD, Child NM, Maredia N. The incremental impact of cardiac MRI on clinical decision-making. Br J Radiol 2016;89:20150662. 26493468 10.1259/bjr.20150662 PMC4985964

[21] Abbasi SA, Ertel A, Shah RV, Dandekar V, Chung J, Bhat G et al Impact of cardiovascular magnetic resonance on management and clinical decision-making in heart failure patients. J Cardiovasc Magn Reson 2013;15:89. 24083836 10.1186/1532-429X-15-89 PMC3851265

[22] Bruder O, Wagner A, Lombardi M, Schwitter J, van Rossum A, Pilz G et al European cardiovascular Magnetic Resonance (EuroCMR) registry–multi national results from 57 centers in 15 countries. J Cardiovasc Magn Reson 2013;15:9. 23331632 10.1186/1532-429X-15-9 PMC3564740

[23] Asher A, Ghelani R, Thornton G, Rathod K, Jones D, Wragg A et al UK perspective on the changing landscape of non-invasive cardiac testing. Open Heart 2019;6:e001186. 31908814 10.1136/openhrt-2019-001186 PMC6927513

[24] Bernhardt P, Steffens M, Kleinertz K, Morell R, Budde R, Leischik R et al Safety of adenosine stress magnetic resonance imaging using a mobile cardiac magnetic resonance system. J Cardiovasc Magn Reson 2006;8:475–8. 16755834 10.1080/10976640600575270

[25] Ge Y, Pandya A, Steel K, Bingham S, Jerosch-Herold M, Chen YY et al Cost-effectiveness analysis of stress cardiovascular magnetic resonance imaging for stable chest pain syndromes. JACC Cardiovasc Imaging 2020;13:1505–17. 32417337 10.1016/j.jcmg.2020.02.029

[26] Walker S, Girardin F, McKenna C, Ball SG, Nixon J, Plein S et al Cost-effectiveness of cardiovascular magnetic resonance in the diagnosis of coronary heart disease: an economic evaluation using data from the CE-MARC study. Heart 2013;99:873–81. 23591668 10.1136/heartjnl-2013-303624

[27] Kozor R, Walker S, Parkinson B, Younger J, Hamilton-Craig C, Selvanayagam JB et al Cost-effectiveness of cardiovascular magnetic resonance in diagnosing coronary artery disease in the Australian health care system. Heart Lung Circ 2021;30:380–7. 32863111 10.1016/j.hlc.2020.07.008

[28] Pandya A, Yu Y-J, Ge Y, Nagel E, Kwong RY, Bakar RA et al Evidence-based cardiovascular magnetic resonance cost-effectiveness calculator for the detection of significant coronary artery disease. J Cardiovasc Magn Reson 2022;24:1. 34986851 10.1186/s12968-021-00833-1 PMC8734365

[29] Arbelo E, Protonotarios A, Gimeno JR, Arbustini E, Barriales-Villa R, Basso C et al 2023 ESC guidelines for the management of cardiomyopathies: developed by the task force on the management of cardiomyopathies of the European Society of Cardiology (ESC). Eur Heart J 2023;44:3503–626. 37622657 10.1093/eurheartj/ehad194

[30] Nguyen ET, Ordovas K, Herbst P, Kozor R, Ng M-Y, Natale L et al Competency based curriculum for cardiovascular magnetic resonance: a position statement of the Society for Cardiovascular Magnetic Resonance. J Cardiovasc Magn Reson 2024;26:100006. 38215698 10.1016/j.jocmr.2023.100006 PMC11211229

[31] Westwood M, Almeida AG, Barbato E, Delgado V, Dellegrottaglie S, Fox KF et al Competency-based cardiac imaging for patient-centred care. A statement of the European Society of Cardiology (ESC). With the contribution of the European Association of Cardiovascular Imaging (EACVI), and the support of the Association of Cardiovascular Nursing & Allied Professions (ACNAP), the Association for Acute CardioVascular Care (ACVC), the European Association of Preventive Cardiology (EAPC), the European Association of Percutaneous Cardiovascular Interventions (EAPCI), the European Heart Rhythm Association (EHRA), and the Heart Failure Association (HFA) of the ESC. Eur Heart J Cardiovasc Imaging 2023;24:1415–24. 37622662 10.1093/ehjci/jead216 PMC10610731

