# Supplementary material for: Awareness of strengths and weaknesses of cardiovascular magnetic resonance imaging: results from a questionnaire survey
Source: Eur Heart J Imaging Methods Pract. 2024 May 23;2(1):qyae050. doi: 10.1093/ehjimp/qyae050 (PMC11472751; doi:10.1093/ehjimp/qyae050)
Supplement: qyae050_Supplementary_Data [file qyae050_Supplementary_Data.docx]

|  | **Entire cohort (n=190)** | **Participants involved in direct patient care (n=39)** | **Participants not involved in direct patient care (n=144)** | **p-value** |
| --- | --- | --- | --- | --- |
| **Age** (years) | 41 (±14) | 45 (±15) | 39 (±14) | 0.078 |
| **Sex** |  |  |  |  |
| Male | 87 (48%) | 13 (33%) | 69 (50%) | 0.06 |
| Female | 95 (52%) | 26 (67%) | 68 (50%) |  |
| **Health Insurance** |  |  |  |  |
| Statutory health insurance | 142 (85%) | 30 (88%) | 109 (85%) | 0.8 |
| Private health insurance | 26 (15%) | 4 (12%) | 19 (15%) |  |
| **Profession** |  |  |  |  |
| Medical Staff | 39 (21%) | 39 (100%) | - | - |
| Administration | 31 (17%) | - | 31 (22%) |  |
| Industry | 27 (15%) | - | 27 (19%) |  |
| IT | 12 (6.6%) | - | 12 (8.3%) |  |
| Health insurance company | 8 (4.4%) | - | 8 (5.6%) |  |
| Politics | 7 (3.8%) | - | 7 (4.9%) |  |
| Health association | 7 (3.8%) | - | 7 (4.9%) |  |
| Other professions | 52 (28%) | - | 52 (36%) |  |
| Not indicated | 7 (3.8%) | - | - |  |

**Table S1:** Demographic characteristics of the entire cohort, as well as subgroups containing participants involved in direct patient care and participants not involved in direct patient care. Participants not reporting an occupation (n=7) were included in the entire cohort but not in any of the subgroups. Continuous data are presented as mean with standard deviation (SD). Categorical data are presented as counts with corresponding percentages. P-values are derived from comparisons between subgroups.

| **Question** | **Entire cohort (n=190)** | **Participants involved in direct patient care (n=39)** | **Participants not involved in direct patient care (n=144)** | **p-value** |
| --- | --- | --- | --- | --- |
| **Which of the following is the leading cause of death in Germany?** | | | | |
| Breast Cancer | 2 (1.1%) | 0 (0%) | 1 (0.7%) | >0.9 |
| Respiratory Diseases | 1 (0.5%) | 0 (0%) | 1 (0.7%) | >0.9 |
| *Cardiovascular Diseases* | 178 (96.2%) | 38 (100%) | 136 (95.8%) | 0.3 |
| Dementia | 0 (0%) | 0 (0%) | 0 (0%) | >0.9 |
| Intestinal Cancer | 4 (2.2%) | 0 (0%) | 3 (2.1%) | >0.9 |
| **Which of the following causes the highest annual health care expenditure in Germany?** | | | | |
| Cancer | 52 (27.8%) | 4 (10.3%) | 48 (33.8%) | **0.004** |
| *Cardiovascular Diseases* | 127 (67.9%) | 34 (87.2%) | 89 (62.7%) | **0.004** |
| Respiratory Diseases | 8 (4.3%) | 1 (2.6%) | 5 (3.5%) | >0.9 |
| **Which of the following examinations exposes patients to radiation?** (Multiple answers allowed) | | | | |
| *Cardiac CT* | 132 (71.4%) | 32 (84.2%) | 96 (68.1%) | 0.051 |
| Cardiac MRI | 46 (24.9%) | 7 (18.4%) | 39 (27.7%) | 0.2 |
| Cardiac Ultrasound / Echocardiography | 30 (16.2%) | 1 (2.6%) | 29 (20.6%) | **0.009** |
| *Cardiac Scintigraphy* | 84 (45.4%) | 23 (60.5%) | 57 (40.4%) | **0.027** |
| *Cardiac Catheterization* | 60 (32.4%) | 19 (50.0%) | 38 (27.0%) | **0.007** |
| **What is the maximum body weight up to which a cardiac MRI exam can technically be carried out using modern devices?** | | | | |
| 80kg | 2 (1.1%) | 0 (0%) | 2 (1.4%) | >0.9 |
| 100kg | 12 (6.4%) | 1 (2.6%) | 11 (7.7%) | 0.5 |
| 125kg | 44 (23.4%) | 8 (21.1%) | 33 (23.1%) | 0.8 |
| 150kg | 76 (40.4%) | 19 (50.0%) | 53 (37.1%) | 0.15 |
| *>180kg* | 54 (28.7%) | 10 (26.3%) | 44 (30.8%) | 0.6 |
| **How long does a cardiac MRI exam usually take?** | | | | |
| 10-20 minutes | 80 (44.2%) | 13 (34.2%) | 66 (47.8%) | 0.14 |
| 30 minutes | 38 (21.0%) | 6 (15.8%) | 30 (21.7%) | 0.4 |
| 60 minutes | 10 (5.5%) | 3 (7.9%) | 7 (5.1%) | 0.5 |
| *Variable (15-60 minutes)* | 53 (29.3%) | 16 (42.1%) | 35 (25.4%) | **0.044** |
| **Which medication is mandatory during a cardiac MRI exam? (**Multiple answers allowed) | | | | |
| *None* | 117 (63.6%) | 26 (68.4%) | 87 (62.1%) | 0.5 |
| Contrast Agent | 74 (40.2%) | 16 (42.1%) | 57 (40.7%) | 0.9 |
| Pharmaceutical Stress Agent | 16 (8.7%) | 4 (10.5%) | 11 (7.9%) | 0.5 |
| Pain Killers | 3 (1.6%) | 0 (0%) | 3 (2.1%) | >0.9 |
| Tranquilizers | 31 (16.8%) | 8 (21.1%) | 20 (14.3%) | 0.3 |
| **Who should carry out and evaluate a cardiac MRI exam?** | | | | |
| Only Radiologists | 41 (22.7%) | 9 (24.3%) | 30 (21.7%) | 0.7 |
| Only Cardiologists | 61 (33.7%) | 11 (29.7%) | 46 (33.3%) | 0.7 |
| *Not important if examiner is board certified* | 82 (45.3%) | 17 (45.9%) | 65 (47.1%) | 0.9 |
| **Which of the following is a / are valid indication(s) for a cardiac MRI examination?**  (Multiple answers allowed) | | | | |
| *Suspected perfusion deficit of the heart muscle* | 137 (74.1%) | 28 (73.7%) | 103 (73.0%) | >0.9 |
| *Unclear thickening of the heart muscle* | 160 (86.5%) | 34 (89.5%) | 120 (85.1%) | 0.5 |
| *Impaired pumping function / Weakness of the heart* | 114 (61.6%) | 22 (57.9%) | 87 (61.7%) | 0.7 |
| *Infections / Inflammation of the heart muscle* | 110 (59.5%) | 26 (68.4%) | 81 (57.4%) | 0.2 |
| *Evaluation before surgery / Evaluation of surgical risk* | 106 (57.3%) | 21 (55.3%) | 82 (58.2%) | 0.7 |
| *Inherited diseases of the heart muscle (“Cardiomyopathies”)* | 120 (64.9%) | 28 (73.7%) | 88 (62.4%) | 0.2 |
| *Congenital heart diseases* | 134 (72.4%) | 28 (73.7%) | 103 (73.0%) | >0.9 |
| **Which of the following factors would you consider as advantages of cardiac MRI?**  (Multiple answers allowed) | | | | |
| *No radiation exposure* | 137 (73.7%) | 31 (79.5%) | 100 (70.9%) | 0.3 |
| *Cost-effectiveness* | 17 (9.1%) | 1 (2.6%) | 13 (9.2%) | 0.3 |
| Easily available | 41 (22.0%) | 5 (12.8%) | 33 (23.4%) | 0.2 |
| *Rare allergic reactions to contrast agents* | 78 (41.9%) | 22 (56.4%) | 53 (37.6%) | **0.035** |
| *Good image quality / informative value, independent of the patient’s constitution* | 128 (68.8%) | 33 (84.6%) | 91 (64.5%) | **0.017** |
| *High temporal and spatial resolution* | 109 (58.6%) | 22 (56.4%) | 53 (37.6%) | 0.8 |
| *Reproducible, regardless of the examiner’s experience* | 62 (33.3%) | 12 (30.8%) | 49 (34.8%) | 0.6 |
| **How would you rate the predictive power of a suspicious stress perfusion cardiac MRI for a future heart attack in comparison to a suspicious stress echocardiogram?** | | | | |
| *Cardiac MRI is better* | 128 (69.2%) | 32 (84.2%) | 91 (64.5%) | **0.02** |
| Same | 21 (11.4%) | 2 (5.3%) | 19 (13.5%) | 0.3 |
| Cardiac MRI is worse | 3 (1.6%) | 0 (0.0%) | 3 (2.1%) | >0.9 |
| I do not know | 33 (17.8%) | 4 (10.5%) | 28 (19.9%) | 0.2 |
| **How would you rate the diagnostic accuracy of cardiac MRI for coronary artery disease compared to a myocardial perfusion scintigraphy?** | | | | |
| *Cardiac MRI is better* | 73 (39.7%) | 18 (46.2%) | 53 (38.1%) | 0.4 |
| Same | 46 (25.0%) | 12 (30.8%) | 31 (22.3%) | 0.3 |
| Cardiac MRI is worse | 4 (2.2%) | 0 (0%) | 4 (2.9%) | 0.6 |
| I do not know | 61 (33.2%) | 9 (23.1%) | 51 (36.7%) | 0.11 |
| **In how many cases do the results from a cardiac MRI exam have an influence on the therapy of the patient?** | | | | |
| Never | 14 (7.5%) | 4 (10.5%) | 9 (6.3%) | 0.5 |
| In 20% of the cases (every 5th examination) | 42 (22.6%) | 9 (23.7%) | 31 (21.8%) | 0.8 |
| In 30% of the cases | 36 (19.4%) | 6 (15.8%) | 30 (21.1%) | 0.5 |
| *In 50% of the cases* | 52 (28.0%) | 8 (21.1%) | 43 (30.3%) | 0.3 |
| In 70% of the cases | 42 (22.6%) | 11 (28.9%) | 29 (20.4%) | 0.3 |
| **Which health insurance currently reimburses the costs for a cardiac MRI exam in Germany?** | | | | |
| *Only private health insurance companies* | 36 (20.0%) | 6 (16.7%) | 27 (19.6%) | 0.7 |
| Only statutory health insurance companies | 16 (8.9%) | 6 (16.7%) | 9 (6.5%) | 0.088 |
| Both | 128 (71.1%) | 24 (66.7%) | 102 (73.9%) | 0.4 |
| **How would you assess the development of current demand for cardiac MRI?** | | | | |
| Decreasing | 0 (0%) | 0 (0%) | 0 (0%) | - |
| Constant | 17 (9.2%) | 2 (5.1%) | 14 (10.1%) | 0.5 |
|  | 151 (82.1%) | 28 (71.8%) | 119 (85.6%) | **0.044** |
| Annual increase around 50% | 16 (8.7%) | 9 (23.1%) | 6 (4.3%) | **<0.001** |
| **How far (from your hometown) would you be willing to travel to obtain a cardiac MRI exam?**  (Multiple answers allowed) | | | | |
| Only if it is available in my hometown | 25 (13.2%) | 5 (12.8%) | 18 (12.5%) | >0.9 |
| Up to 10 km | 8 (4.2%) | 2 (5.1%) | 6 (4.2%) | 0.7 |
| Up to 25-50 km | 85 (44.7%) | 18 (46.2%) | 61 (42.4%) | 0.7 |
| Up to 100 km | 62 (32.6%) | 12 (30.8%) | 50 (34.7%) | 0.6 |
| I would prefer a mobile cardiac MRI exam (exam in my hometown and evaluation by experts via telemedicine) | 33 (17.4%) | 7 (17.9%) | 24 (16.7%) | 0.8 |
| **For a radiation-free cardiac check-up, I would be willing to contribute the following amount:** | | | | |
| 75 Euro | 68 (40.7%) | 15 (42.9%) | 50 (39.7%) | 0.7 |
| 125 Euro | 54 (32.3%) | 13 (37.1%) | 39 (31.0%) | 0.5 |
| 250 Euro | 34 (20.4%) | 5 (14.3%) | 28 (22.2%) | 0.3 |
| 500 Euro | 9 (5.4%) | 2 (5.7%) | 7 (5.6%) | >0.9 |
| 1000 Euro | 2 (1.2%) | 0 (0%) | 2 (1.6%) | >0.9 |

**Table S2:** Overview of answers to selected questions for the entire cohort, as well as subgroups containing participants involved in direct patient care and participants not involved in direct patient care. Participants not reporting an occupation (n=7) were included in the entire cohort but not in any of the subgroups. The frequency of given answers is provided as count with corresponding percentage of evaluable replies within the respective (sub-)group. Correct answers (if applicable) are written in italics. P-values are derived from comparisons of the two subgroups. P-values indicating statistical significance (p<0.05) are highlighted in bold.

**Figure S1:** Knowledge and Awareness of Indications **(A+C)** and Therapeutic Impact **(B+D)** of Cardiac MRI among the entire cohort **(A+B)** compared between the “participants involved in direct patient care” (dark red) and “participants not involved in direct patient care” (light red) subgroups **(C+D)**.

**Figure S2:** Participants’ willingness to travel **(A+C)** and financially contribute **(B+D)** to obtain a cardiac MRI examination. Results are given for the entire cohort **(A+B)** compared between the “participants involved in direct patient care” (dark red) and “participants not involved in direct patient care” (light red) subgroups **(C+D)**.
